# Nanostructured surface topographies have an effect on bactericidal activity

**DOI:** 10.1186/s12951-018-0347-0

**Published:** 2018-02-28

**Authors:** Songmei Wu, Flavia Zuber, Katharina Maniura-Weber, Juergen Brugger, Qun Ren

**Affiliations:** 1 School of Science, Beijing Jiaotong University, No. 3 Shangyuancun, Haidian District, Beijing, 100044 People’s Republic of China; 2 Laboratory for Biointerfaces, Empa, Swiss Federal Laboratories for Materials Science and Technology, Lerchenfeldstrasse 5, 9014 St. Gallen, Switzerland; 3 Microsystems Laboratory, École Polytechnique Fédérale de Lausanne, Station 17, 1015 Lausanne, Switzerland

**Keywords:** Antibacterial surface, Nanostructure, Nanoscale topography, Bactericidal activity

## Abstract

**Background:**

Due to the increased emergence of antimicrobial resistance, alternatives to minimize the usage of antibiotics become attractive solutions. Biophysical manipulation of material surface topography to prevent bacterial adhesion is one promising approach. To this end, it is essential to understand the relationship between surface topographical features and bactericidal properties in order to develop antibacterial surfaces.

**Results:**

In this work a systematic study of topographical effects on bactericidal activity of nanostructured surfaces is presented. Nanostructured Ormostamp polymer surfaces are fabricated by nano-replication technology using nanoporous templates resulting in 80-nm diameter nanopillars. Six Ormostamp surfaces with nanopillar arrays of various nanopillar densities and heights are obtained by modifying the nanoporous template. The surface roughness ranges from 3.1 to 39.1 nm for the different pillar area parameters. A Gram-positive bacterium, *Staphylococcus aureus*, is used as the model bacterial strain. An average pillar density at ~ 40 pillars μm^−2^ with surface roughness of 39.1 nm possesses the highest bactericidal efficiency being close to 100% compared with 20% of the flat control samples. High density structures at ~ 70 pillars μm^−2^ and low density structures at < 20 pillars μm^−2^ with surface roughness smaller than 20 nm reduce the bactericidal efficiency to almost the level of the control samples.

**Conclusion:**

The results obtained here suggests that the topographical effects including pillar density and pillar height inhomogeneity may have significant impacts on adhering pattern and stretching degree of bacterial cell membrane. A biophysical model is prepared to interpret the morphological changes of bacteria on these nanostructures.

**Electronic supplementary material:**

The online version of this article (10.1186/s12951-018-0347-0) contains supplementary material, which is available to authorized users.

## Background

Antibacterial materials are important for various industrial applications. For example, antibacterial surfaces are highly desired in biomedical applications to prevent the adhesion of viable pathogenic bacteria on material surfaces that could proliferate to drug-resistant biofilms and cause chronic infections. Conventional biochemical approaches rely on the coatings with biocidal agents and drugs such as silver and antibiotics. With increased level of antimicrobial resistance, it is today important to develop novel efficient antibacterial strategies to prevent bacterial adhesion and proliferation on material surfaces [1–4].

In the past decade, the research on antibacterial effects using micro/nano scale topographies has become a focus topic. There are in principle two strategies to reduce surface microbial contamination, either by adhesion prevention or alternatively by contact killing. Up to date, various microscale topographies such as ridges, ripples and grooves have been demonstrated to prevent or reduce bacterial adhesion and biofilm formation on the surface [5–14]. Recently, nanopillar structures on *Psaltoda claripennis* cicada wings were found to be lethal for *Pseudomonas aeruginosa*, a Gram-negative bacterial strain [15]. Therein the bactericidal property was proposed to be a pure mechanical effect as a consequence of the physical interactions between the nanoscale topography of the substrate and the adhering pathogenic cells [16]. The adsorbed bacterial cell membranes were suspended between the nanopillars, thereby the cell membrane was deformed, stretched and torn apart, leading to eventual cell death. It was later demonstrated that these wing surfaces were only effective for Gram-negative bacterial strains, as Gram-positive cells with thicker cell walls are generally more rigid and withstand larger mechanical deformation [17].

Inspired by the excellent bactericidal surface structures in nature, various surfaces with nanoscale protrusions have been successfully fabricated. Among them, several materials such as black Si [18, 19] and titanium [20] or titania nanowires [21] exhibited efficient biocidal effects against both Gram-positive and negative bacterial strains. There are also surface nanopillar structures with pillar diameters comparable to those on the cicada’s wing, however only a minor or negligible biocidal effect could be demonstrated [20, 22]. Recently, it was reported that the surface nanopillar-array structures of several cicada species showed significant difference in bactericidal activity [23–25]. These observations underline the importance of better understanding the relationship between surface topographical features and bactericidal properties.

In this paper we present a systematic study of topographical effect on bactericidal activity of nanostructured surfaces. Nanostructured Ormostamp polymer surfaces were fabricated by nano-replication technology using nanoporous templates to obtain pillar shapes, with various morphologies, densities and aspect ratios. The polymer nanostructures were evaluated for their bactericidal activity against *Staphylococcus aureus* (ATCC 6538). It is a Gram-positive pathogen isolated from human lesion which in general causes a broad spectrum of infections, including wound infections, food poisoning, meningitis, bacteremia and many others. Since Gram-positive cells with thicker cell walls are generally more resistant to mechanical deformation compared with the Gram-negative strains, the Gram-positive *S. aureus* strain was selected in order to characterize the bactericidal efficiency of the nanostructured surfaces. Based on the obtained results, a biophysical model was provided to interpret the morphological changes of bacteria on these nanostructures.

## Methods

### Fabrication of nanostructured Ormostamp surfaces

Nanometer-scale pillar structures on the Ormostamp polymer material were fabricated by UV nano-replication technology, which is based on pattern transfer of a nanopore template structure into a UV-curable material. The fabrication process and schematic templates are illustrated in Additional file 1: Fig. S1a–c. Thin nanoporous templates were developed using Al anodization and deep reactive etching techniques in our early work [26]. Briefly, 150 nm Al thin film was at first deposited on Si wafer. A vertical alumina nanoporous layer with thickness of 200 nm was then generated by the electrochemical etching of aluminum thin film in acidic solution. The nanopores were organized in a near-hexagonal pattern with inhomogeneous pore size and pore depth. Afterwards, deep reactive etching of Si was performed by using the top nanopore alumina layer as mask. Consequently, nanopore structures were transferred into the underlying Si substrate. For example, one typical nanopore template is shown in Additional file 1: Fig. S1b, where deep and shallow pores are indicated. There are also some pore structures in the top layer which were not etched into Si, leading to reduced pore density in the Si layer. By controlling the electrochemical etching and deep reactive etching conditions, the diameter, density and depth of the nanopores can be modulated. The schematics of six nanoporous templates are shown in Additional file 1: Fig. S1c. The material OrmoStamp^®^ used for nanoimprint was purchased from Micro Resist Technology GmbH. The nanopillar structures were transferred onto glass substrates of 1 × 1 cm^2^. For each structure, 4–8 samples were prepared per batch.

### Scanning electron microscope and atomic force microscope

SEM images were taken using a field-emission SEM (Zeiss LEO 1550) at 1 kV under 20, 30 and 50 k magnification with stage angle of 0° and 30°. AFM images were taken by using Nanosurf Flex-Axiom setup with tip of Tap190Al-G (190 kHz, 48 N m^−1^).

### Bacteria culture and adhesion on surfaces

*Staphylococcus aureus* used in this study were purchased from ATCC (ATCC 6538). Bacteria from glycerol stocks were cultivated on Tryptic Soy agar plates (Sigma Aldrich). A single colony was transferred to 10 mL of tryptic soy broth (TSB) and incubated overnight at 37 °C. 1 mL of the overnight culture was added to 10 mL fresh TSB and incubated until it reached late exponential growth phase. The suspension was diluted with 0.9% NaCl to approximate 10^5^ colony forming units (CFU) mL^−1^. 50 µL of the diluted suspension was loaded to the sterilized Ormostamp samples (three repeats per sample) deposited in 12-well plates and incubated for 2 h at room temperature. The cell suspension was removed and the surfaces were washed three times with 1 mL 0.9% NaCl to remove non-adhered cells. Bacteria on the surfaces were then investigated using fluorescence microscopy after staining.

### Bacteria viability analysis using live-dead staining and fluorescence microscopy

A mixture of 2.5 μM SYTO9 (Life technologies) and 15 µM propidium iodide (PI) (Sigma) in 0.9% NaCl solution was freshly prepared according to manufacturer’s instruction and used to stain bacterial cells as described previously [27, 28]. 50 µL of the mixture was added to the top of the sample in a microplate well and the plate was incubated for 30 min at room temperature in the dark. The staining mixture was then removed and the wells with samples were washed three times with 1 mL ddH_2_O. The samples were then analyzed using a fluorescence microscope (Leica DM6000B). The laser was used at 488 nm for excitation, and the emission was observed at 528 nm (SYTO9) and 645 nm (PI), respectively. For each sample, two independent experiments with three images per sample per experiment were performed. Images were taken at the fixed locations on each sample to obtain a statistical overview.

## Results and discussion

### Characterization of nanostructured polymer surfaces

**Fig. 1:**
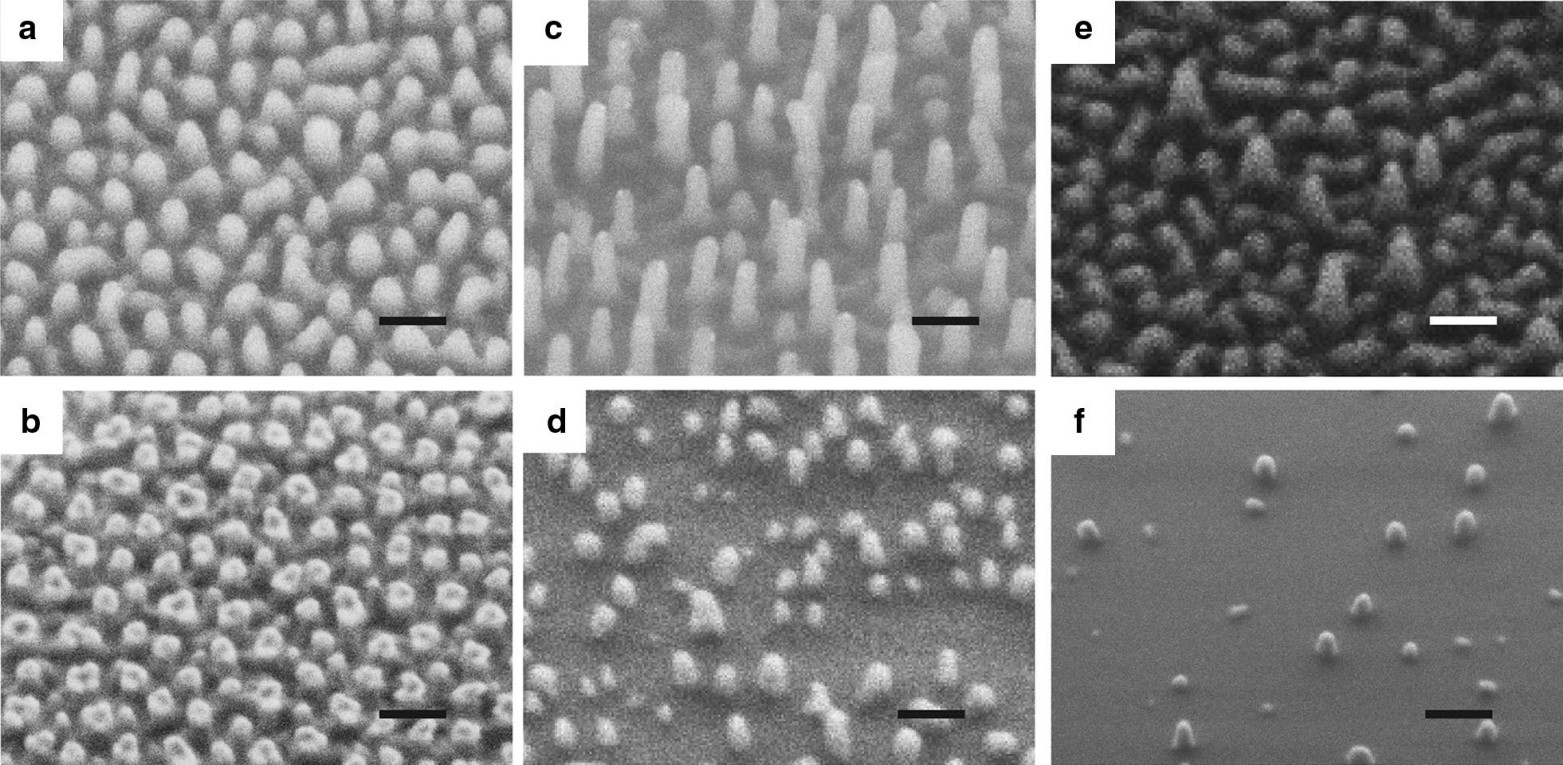
SEM images of nanostructured Ormostamp surfaces S(a)–S(f) (scale bar: 200 nm). Typical surface structures show nanoscale pillar-like protrusions. The images from left to right columns correspond to high to low pillar density, while those in up and down rows represent large to small pillar height. The structural features of S(a)–S(f) are summarized in Table 1

**Fig. 2:**
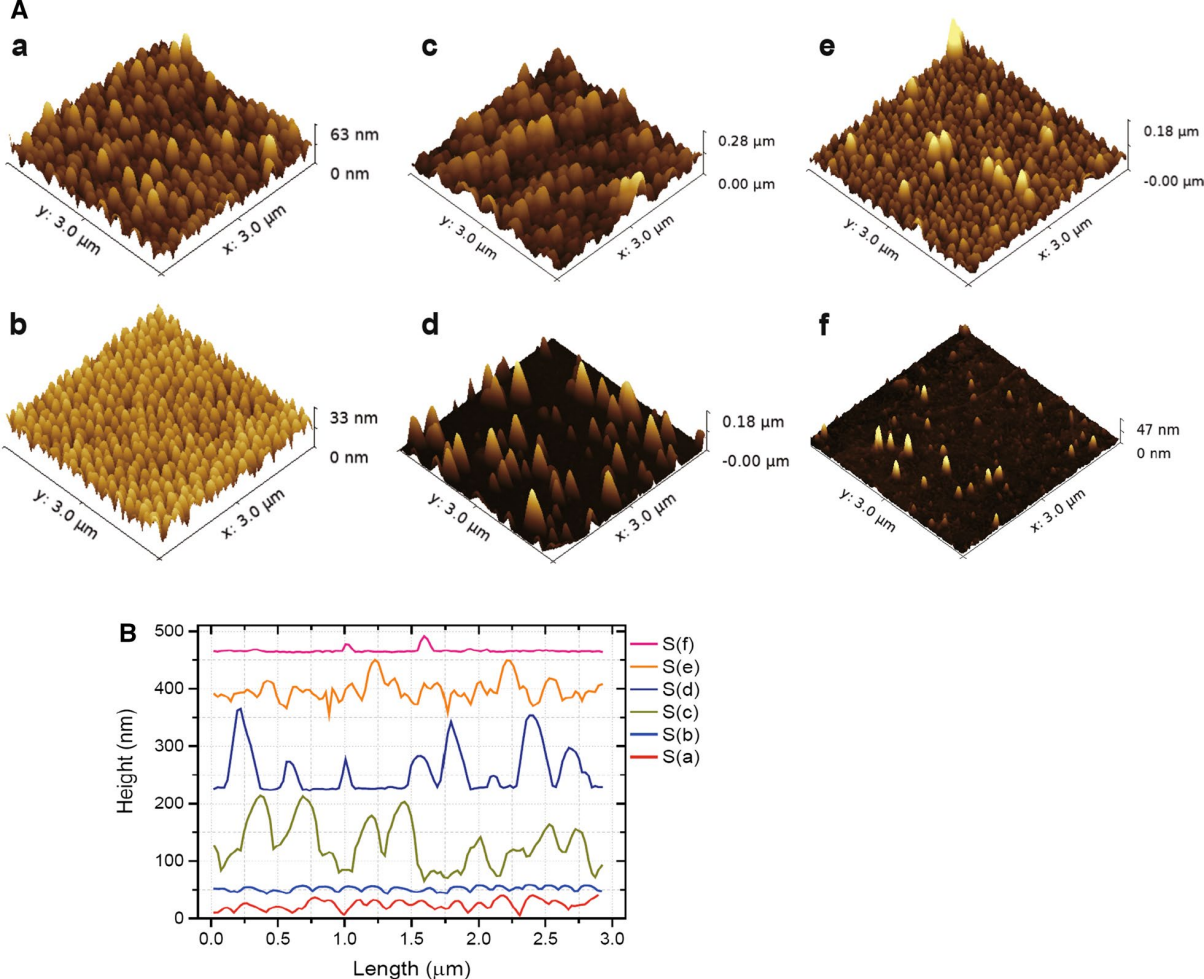
**A** AFM images of nanostructured Ormostamp surfaces **a**–**f** corresponding to the structures S(a)–S(f) shown in Fig. 1. **B** Profiles of nanostructured Ormostamp surfaces **a**–**f** extracted from AFM images

The SEM images of the 6 fabricated nanostructured Ormostamp surfaces are presented in 3 columns and 2 rows in Fig. 1. The structures from left to right columns correspond to high to low pillar density, while those in up and down rows have large to small pillar height. The typical nanopillars shown in Fig. 1a, b have vertical side walls with diameter of ~ 80 nm and average density at ~ 70 pillars μm^−2^. The morphology, density and height of the nanopillar structures can be modulated by modifying the nanoporous template. The nanopillar structures in Fig. 1c–f have conical shapes and heterogeneous heights. For example, there are both high (> 200 nm) and low pillars (< 200 nm) in Fig. 1c. The structures in Fig. 1d are 200 nm shorter that those shown in Fig. 1c, thereby those nanopillars which are lower than 200 nm in Fig. 1c disappear on the samples shown in Fig. 1d. Considering the number of pillars which are higher than 200 nm in structure c, the average pillar densities of structure c and d are similar at ~ 40 pillars μm^−2^. For structure e, the number of high pillars (> 200 nm) are much less than that in structure c. Considering the number of pillars which are higher than 200 nm in structure e, the average pillar densities of structure e and f are less than 20 pillars μm^−2^. The topographical features of these nanostructures are shown in AFM images (Fig. 2A) and the extracted profiles are summarized in Fig. 2B. It can be seen that the pillar height is homogeneous for samples a and b with pillar density at ~ 70 pillars μm^−2^ and root mean square surface roughness smaller than 10 nm. For samples c–e with lower pillar densities, the pillar height difference ∆h ranges from 10 to 50 nm, leading to increased surface roughness. The sample f has smallest surface roughness at 3.1 nm with lowest pillar density and pillar height. It can also be seen that due to the AFM tip effect, the pillar heights for samples a and b with relatively high pillar density at ~ 70 pillars μm^−2^ are much smaller than the actual values. The tip effect may also lead to sharper caps for conical shaped pillars. The feature size of these nanostructures is summarized in Table 1.

**Table 1:** Summary of feature size and average bactericidal efficiency of nanostructured Ormostamp surfaces S(a) to S(f)

Sample	Pillar height h (nm)	Center to center distance d (nm)	Average pillar density n (# μm^−2^)	RMS roughness (nm)	Bactericidal efficiency (%)
	~ 250	~ 130	~ 68	8.6	31
	~ 200	~ 130	~ 68	4.4	23
	~ 400	~ 170	~ 40	39.1	100
	~ 200	~ 170	~ 40	28.0	98
	~ 300	> 300	< 20	16.9	26
	~ 150	> 300	< 20	3.1	31
	–	–	–	1.6	21

Their feature size was analyzed according to SEM images. Bactericidal efficiency was evaluated based on fluorescence microscopy images

### Bactericidal activities of various nanostructured surfaces

**Fig. 3:**
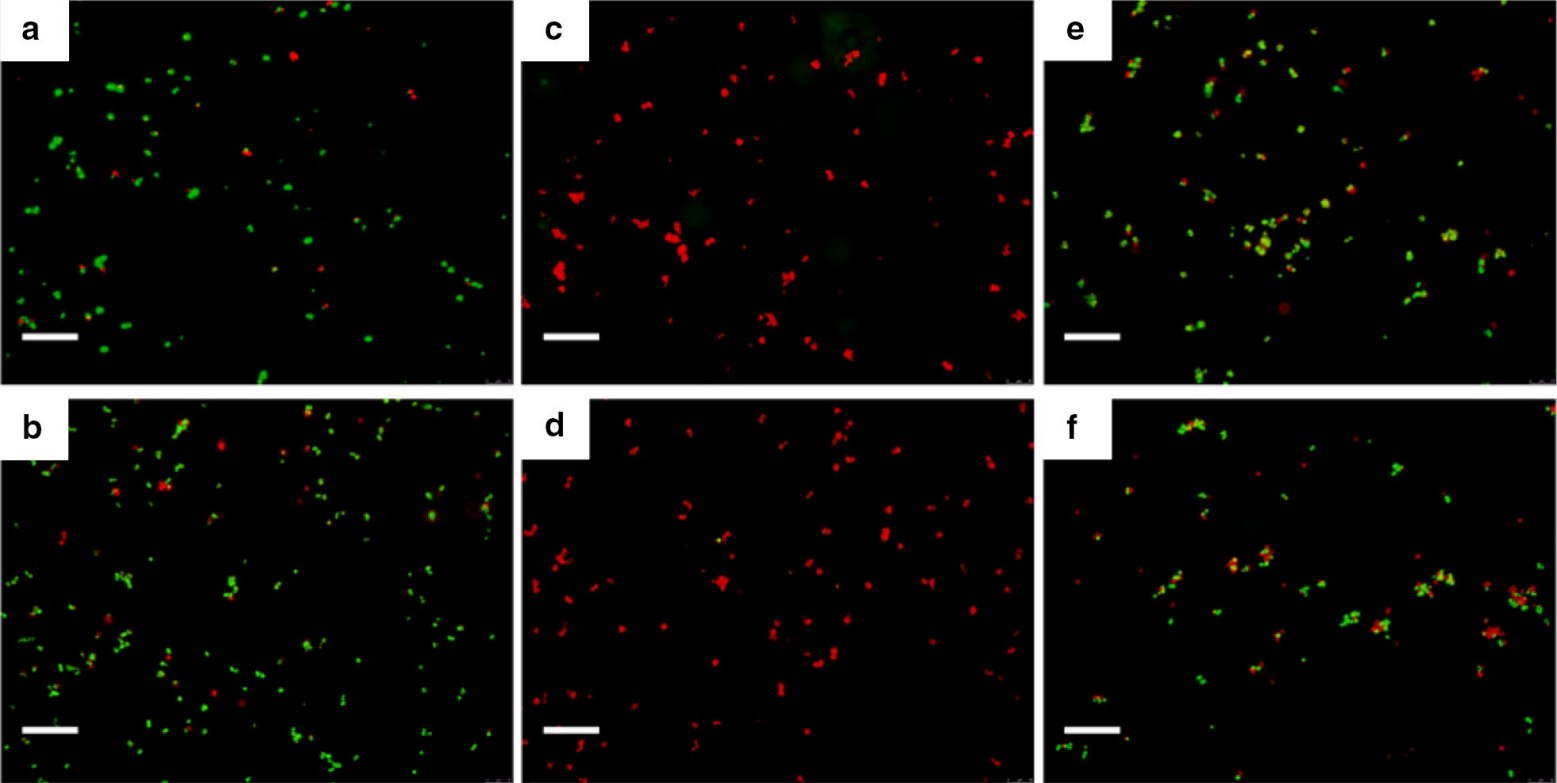
Characterization of *S. aureus* viability on various nanostructured surfaces by fluorescence microscopy after SYTO9/PI staining. Fluorescent images **a**–**f** were obtained on the nanostructured surfaces S(a)–S(f) shown in Fig. 1, respectively. Green and red colors indicate live and dead cells, respectively. Scale bar: 20 μm

**Fig. 4:**
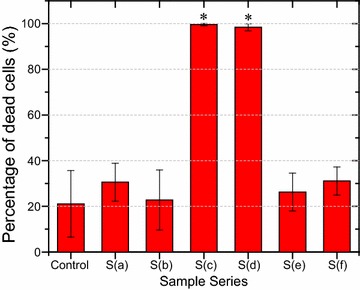
Bactericidal activities of various nanostructured surfaces. Columns are displayed as average percentage of dead cells per imaging field. For each type of surfaces, two independent experiments with three images per sample per experiment were performed. Images were taken at the fixed locations on each sample to obtain a statistical overview. Error bars are shown as standard error for at least three images. The statistical significance was determined for each data set using the unpaired, parametric, two-tailed *t* test. *P < 0.001 vs. the control substrate

The viability of *S. aureus* on various nanostructured surfaces was characterized by fluorescence microscopy after SYTO9/PI staining. Representative images are shown in Fig. 3. Structures c and d with a pillar density at ~ 40 pillars μm^−2^ exhibit the highest bactericidal effect where almost all the bacterial cells appear dead on the surfaces, whereas the other surfaces including the flat control surfaces (Additional file 1: Fig. S2) display much lower bactericidal activity and the majority of the bacterial cells remain alive. By counting the number of living and dead cells per imaging area, the percentage of dead cells on various nanostructured surfaces was calculated and summarized in Fig. 4. The percentage of dead cells reaches almost 100% on structure c and d, while the percentage drops to 20–30% for the rest of nanostructures (a, b, e and f) as well as the flat control surface. As microscopic images have limited view, a proliferation assay was developed in our earlier work in order to evaluate the antibacterial property of the entire surface of a macroscopic sample with area of 1 × 1 cm^2^ [27]. The obtained results based on the proliferation assay, shown in supporting information Additional file 1: Fig. S3, are consistent with the staining observations.

**Fig. 5:**
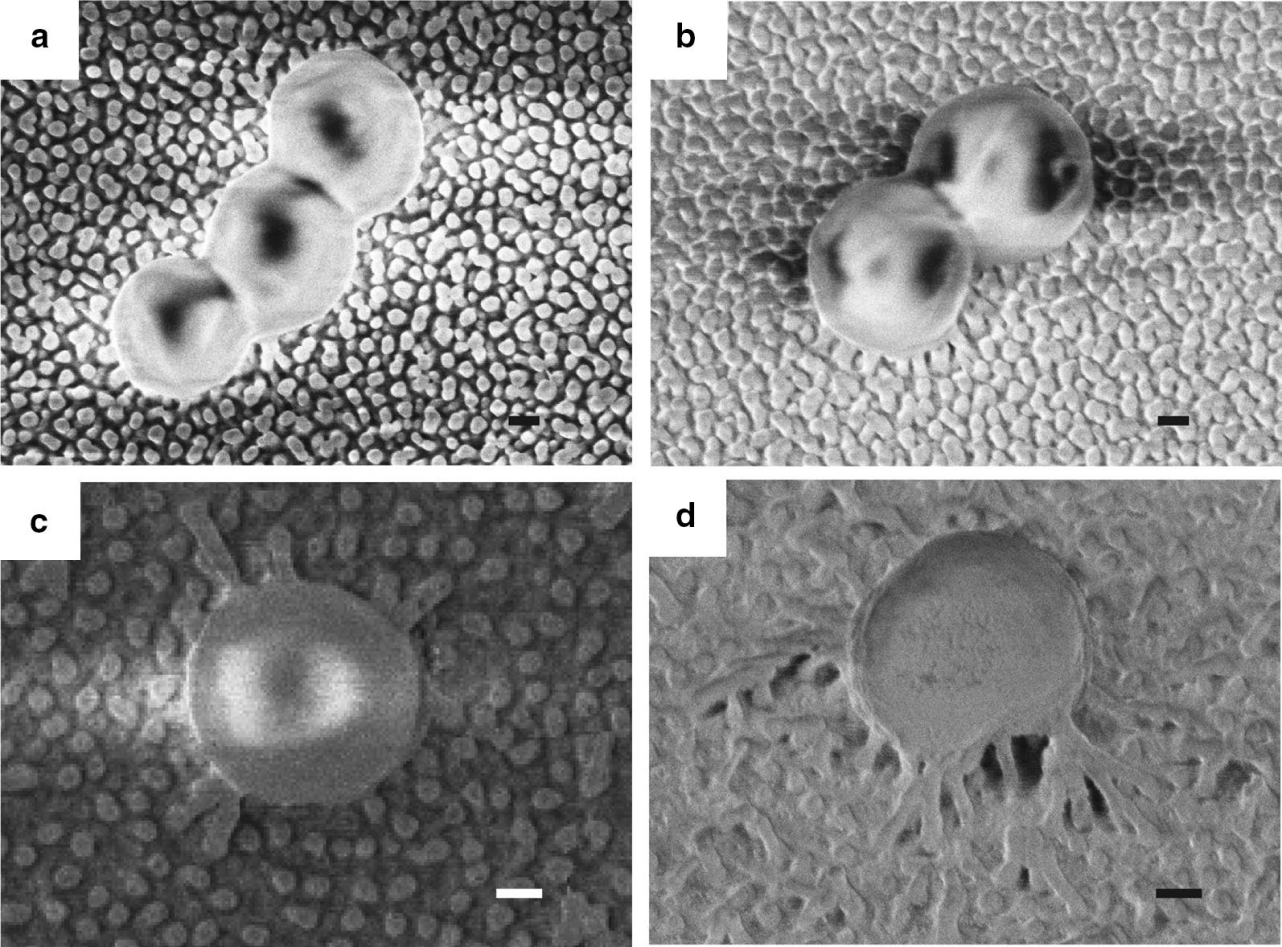
SEM images of *S. aureus* cells on nanostructured Ormostamp surfaces S(b) and S(c): **a**, **b** are top and tilted views on S(b); **c**, **d** are top and tilted views on S(c). Scale bar: 200 nm

The pronounced bactericidal efficiency of the structures c and d can be likely attributed to their appropriate density of nanopillar arrays at ~ 40 pillars μm^−2^. High density at ~ 70 pillars μm^−2^ (structures a, b) and low density at < 20 pillars μm^−2^ surfaces (structures e and f) show only slightly higher cell death ratio than that on flat control samples. It was also observed that the pillar height (150–400 nm in this study) has minor effect on the bactericidal property of nanostructures (Figs. 3, 4). Typical SEM images of *S. aureus* cells on all the samples are shown in Additional file 1: Fig. S4. Although the interaction of bacterial cells with nanopillars is noticed on all the nanostructures, obvious cell deformation can only be observed on structure S(c) and S(d) with effective pillar density of ~ 40 pillars μm^−2^. This observation is in agreement with the experimental results of live/dead staining and proliferation assay. Since all the nanopillars have similar diameters, the bactericidal mechanism is considered to be the stretching of the cell membrane between the nanopillars rather than direct piercing of the cell membrane. Such a membrane distortion is most obvious on the structure S(c) as the nanopillars have highest aspect ratios, which allow for the suspension of the bacterial cells. The comparison of the morphology of *S. aureus* cells on nanopillar structure S(b) and S(c), which possess the lowest and highest bactericidal activity, respectively, analyzed by SEM imaging is detailed in Fig. 5. It can be seen that the bacterial cells maintain their original round shape on the surface S(b) with high pillar density (~ 70 pillars μm^−2^) and small surface roughness (4.4 nm). In contrast, the cell is stretched and deformed on the high pillars and sink onto the entire surface S(c) with lower pillar density (~ 40 pillars μm^−2^) and large surface roughness (39.1 nm). Since there are nearly half of pillars higher than 200 nm and the rest lower than 200 nm on the surface, the cells at first get in contact with and become distorted on the high pillars before they adhere to the entire surface. If the cell stretching goes beyond a critical value, then the cell membrane eventually ruptures. It is likely that similar cell stretching and deformation also occur on surface S(d) which is 200 nm shorter than S(c) with smaller roughness at 28.0 nm, leading to comparably high bactericidal effect. We thus attribute the comparable high bactericidal efficiency for structures S(c) and S(d) to their appropriate effective pillar density and inhomogeneity of pillar height.

### Biophysical model of bacterial cells adhered on nanostructured surfaces

In order to investigate the topographical effect on bactericidal properties of nanostructured features, recent studies focus on various nanopillar structures on insect wing surfaces of several spices of dragonflies and cicadas [23–25] as well as microfabricated biomimetic nanopillar-with-cap structures [22, 29]. These nanopillar structures however vary simultaneously at diameter and spacing, and the resulting bactericidal activities are significantly different. Taking three wing structures of different Cicada species, it was demonstrated that the nanopillars with diameter of 156 nm and density of 45 pillars μm^−2^ have the most efficient bactericidal activity, and the bactericidal efficiency decreases with increasing diameter and spacing of nanopillar arrays [24]. Similar tendency was also observed for poly(methyl methacrylate) (PMMA) nanopillar arrays [29]. Recently, it was also reported that acrylonitrile butadiene styrene (ABS) nanopillars with diameter of ~ 60 nm and interpillar distance of 100 nm (which corresponds to high density of 115 pillars μm^−2^) has no effect on bacterial adhesion and viability for *S. epidermidis* compared with flat control surfaces [22]. This is likely due to very high density of nanopillars so that the stretching degree of cell membrane is limited. In this work, the nanopillars have a diameter of ~ 80 nm, which is around 10 times smaller than the diameter of a *S. aureus* cell. We have observed the highest bactericidal activity with average pillar density of ~ 40 μm^−2^, which is comparable to that of the nanopillar structures on cicada’s wing. These results suggest that the density of the nanostructure plays an important role in the adhesion pattern and therefor the stretching degrees of the attached bacterial cells.

**Fig. 6:**
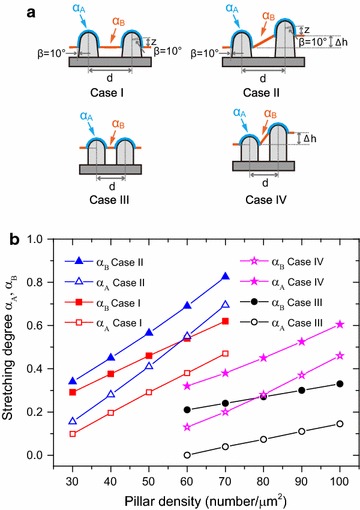
Biophysical model of bacterial cell adhering and stretching on nanostructured surfaces. **a** The bacterial cell was modeled as an elastic membrane adhering on the pillars with the stretching degree α_A_ and suspending between them with the stretching degree α_B_. Two interaction patterns are schematically shown for relatively low pillar density at 30–70 pillars μm^−2^ (Cases I and II) and high pillar density at 60–100 pillars μm^−2^ (Cases III and IV). In Case I, the cell membrane covers the top cap of the nanopillars and also part of the vertical pillar wall marked by vertical distance z; while in Case II, the pillars have inhomogeneous height characterized by ∆h. In Case III, the cell membrane covers only the top cap of the nanopillars; while in Case IV, the dense pillars have height difference ∆h. **b** Calculated equilibrium stretching degrees α_A_ and α_B_ for the four cases at various pillar densities

The bactericidal effect of nanostructures has previously been considered as a pure mechanical effect [16, 30, 31]. The nanostructured surfaces S(c) and S(d) have similar effective feature size to that of *Psaltoda claripennis* cicada wing surfaces with conical nanopillars of 100 nm in diameter at the bottom, 60 nm in diameter at the cap and a center-to-center distance of 170 nm. In the biophysical model described by Pogodin et al. [16], as illustrated in Fig. 6a Case I, the adsorbed bacteria cell was modeled as an elastic membrane adhering on the pillars (with the stretching degree α_A_) and suspending between them (with the stretching degree α_B_). With appropriate pillar density, the cell membrane covers not only the top cap of the pillars but also the surface of vertical pillar walls marked by distance z (in this case 40 nm). Each site of the bacterial cell that is adsorbed on the nanopillars contributes to the energy gain, which is balanced by the free energy loss associated with cell membrane deformation. The equilibrium stretching degrees α_A_ and α_B_ of the membrane as shown in Fig. 6b were obtained based on numerical minimization of the total free energy (details are described in the previous reported work [16] and supporting information Biophysical model). The stretching degree of the cell membrane suspending between the pillars (α_B_) was found to be larger than that adhering on the pillar surface (α_A_). The equilibrium stretching degree α_B_ at 0.38 compared with α_A_ at 0.20 were obtained in the interaction Case I with pillar density at ~ 40 pillars μm^−2^. When the membrane stretching degree α_B_ goes beyond this critical value, the bacterial cell membrane is ruptured. On the nanopillars with different heights, the cell membrane suspending between the nanopillars is further elongated, resulting in larger stretching degree. This effect is illustrated as Case II with the height difference ∆h = 30 nm. The corresponding stretching degree α_B_ increases from 0.38 in Case I to 0.42.

The equilibrium stretching degree depends not only on the structural features but also the interaction pattern of the cell membrane on the nanopillars. As illustrated in Fig. 6a Case I, if the interaction pattern, i.e., the covering depth z can be kept constant, the stretching degrees should certainly increase with higher pillar densities. Such an interaction pattern, however, may not be valid for the case of dense nanopillars. As the smaller distance d between the nanopillars limits the space for cell adsorption on the surface of vertical pillar walls, the covering depth z was supposed to decrease.

We thus suggest the interaction Case III for nanostructures a and b with high pillar density at ~ 70 pillars μm^−2^. The nanopillars have a diameter of 80 nm with vertical walls and half-sphere shaped caps. On the dense nanopillar arrays, the cell membrane covers only the top cap. In this case, the equilibrium stretching degree α_B_ was calculated to be 0.24. This is smaller than that of 0.38 obtained in Case I for the lower pillar density at ~ 40 pillars μm^−2^. Consequently, the bactericidal efficiency is much lower. The stretching degree of the cell membrane is enhanced for nanopillar array with inhomogeneous height ∆h. With the same pillar height difference ∆h = 30 nm at pillar density of ~ 70 pillars μm^−2^, the corresponding stretching degree α_B_ increases from 0.24 in Case III to 0.38 in Case IV.

For the low pillar density surfaces (< 20 pillars μm^−2^), the cell interaction pattern is close to that in Case I but the numbers of stretching centers are much lower. For example, the equilibrium stretching α_B_ of 0.22 with density at 20 pillars μm^−2^ could be extrapolated from the Case I curve in Fig. 6b, which is much smaller than 0.38 for pillar density at ~ 40 pillars μm^−2^. This value is however comparable to 0.24 for pillar density at ~ 70 pillars μm^−2^ in Case III.

In summary, based on the biophysical model, the equilibrium stretching degrees α_B_ for structure c and d with diameter of ~ 80 nm and pillar density at ~ 40 pillars μm^−2^ is larger than that for similar structures with high pillar density at ~ 70 pillars μm^−2^ and low pillar densities < 20 pillars μm^−2^. The theoretical results are in consistence with the experimental observations of topographical effect on bactericidal activities. The density of the nanostructure as well as the height homogeneity and hence the coverage pattern of the cell membrane on the nanopillars is likely the dominating factor in determining stretching degrees of the cell membrane.

## Conclusions

In conclusion, a systematic study on the topographical effect on bactericidal activity of nanostructured surfaces was performed by using nanopillar structures with similar diameters but various densities and heights. The typical nanopillars have a diameter of ~ 80 nm, which is around 10 times smaller than the diameter of the *S. aureus* cell. It was found that the nanostructured surfaces with average pillar density at ~ 40 pillars μm^−2^ and roughness of 39.1 nm possesses the highest bactericidal efficiency against *S. aureus*, whereas high density at ~ 70 pillars μm^−2^ and low density at < 20 pillars μm^−2^ with surface roughness smaller than 20 nm reduce the bactericidal efficiency to almost the level of the control samples. The biophysical model revealed that the equilibrium stretching degrees are highly dependent on the interaction pattern of cell membranes on the nanopillars with different pillar densities and heights. With appropriate pillar density, the cell membrane covers the top cap and also part of the vertical pillar wall, leading to large stretching degree. The stretching degree is further enhanced by the pillar height inhomogeneity. Our nanofabrication technique is based on self-organization and nanoscale replication processes, which allow cost-efficient scalable applications. Our technological platform and systematic study provide potential prospects for further developing functional antibacterial surfaces.

## Additional file

**Additional file 1: Figure S1.** Fabrication of nanostructured Ormostamp surfaces. **Figure S2**. Fluorescence image of *S. aureus* cells on smooth control surface. **Figure S3.** Quantification of bactericidal efficiency by proliferation measuremen. **Figure S4.** SEM images of *S. aureus* cells on nanostructured Ormostamp surfaces S(a)-S(f). **Figure S5.** Biophysical model of bacterial cells adhered on nanostructured surfaces.
