# Supplementary material for: Nanostructured surface topographies have an effect on bactericidal activity
Source: J Nanobiotechnology. 2018 Feb 28;16:20. doi: 10.1186/s12951-018-0347-0 (PMC5830064; doi:10.1186/s12951-018-0347-0)
Supplement: Supplementary file 1 — Additional file 1: Figure S1. Fabrication of nanostructured Ormostamp surfaces. Figure S2. Fluorescence image of S. aureus cells on smooth control surface. Figure S3. Quantification of bactericidal efficiency by proliferation measuremen. Figure S4. SEM images of S. aureus cells on nanostructured Ormostamp surfaces S(a)-S(f). Figure S5. Biophysical model of bacterial cells adhered on nanostructured surfaces. [file 12951_2018_347_MOESM1_ESM.docx]

Additional Information for

**Nanostructured surface topographies have an effect on bactericidal activity**

Songmei Wu ^a^*, Flavia Zuber ^b^, Katharina Maniura-Weber ^b^,

Juergen Brugger ^c^ and Qun Ren ^b^*

^a^ School of Science, Beijing Jiaotong University,

No.3 Shangyuancun, Haidian District, Beijing 100044, P. R. China

^b^ Laboratory for Biointerfaces, Empa, Swiss Federal Laboratories for Materials Science and Technology, Lerchenfeldstrasse 5, CH-9014 St. Gallen, Switzerland.

^c^ Microsystems Laboratory, École Polytechnique Fédérale de Lausanne,
Station 17, 1015 Lausanne, Switzerland

Correspondence: smwu@bjtu.edu.cn, qun.ren@empa.ch

1. Fabrication of nanostructured polymer surfaces.

*
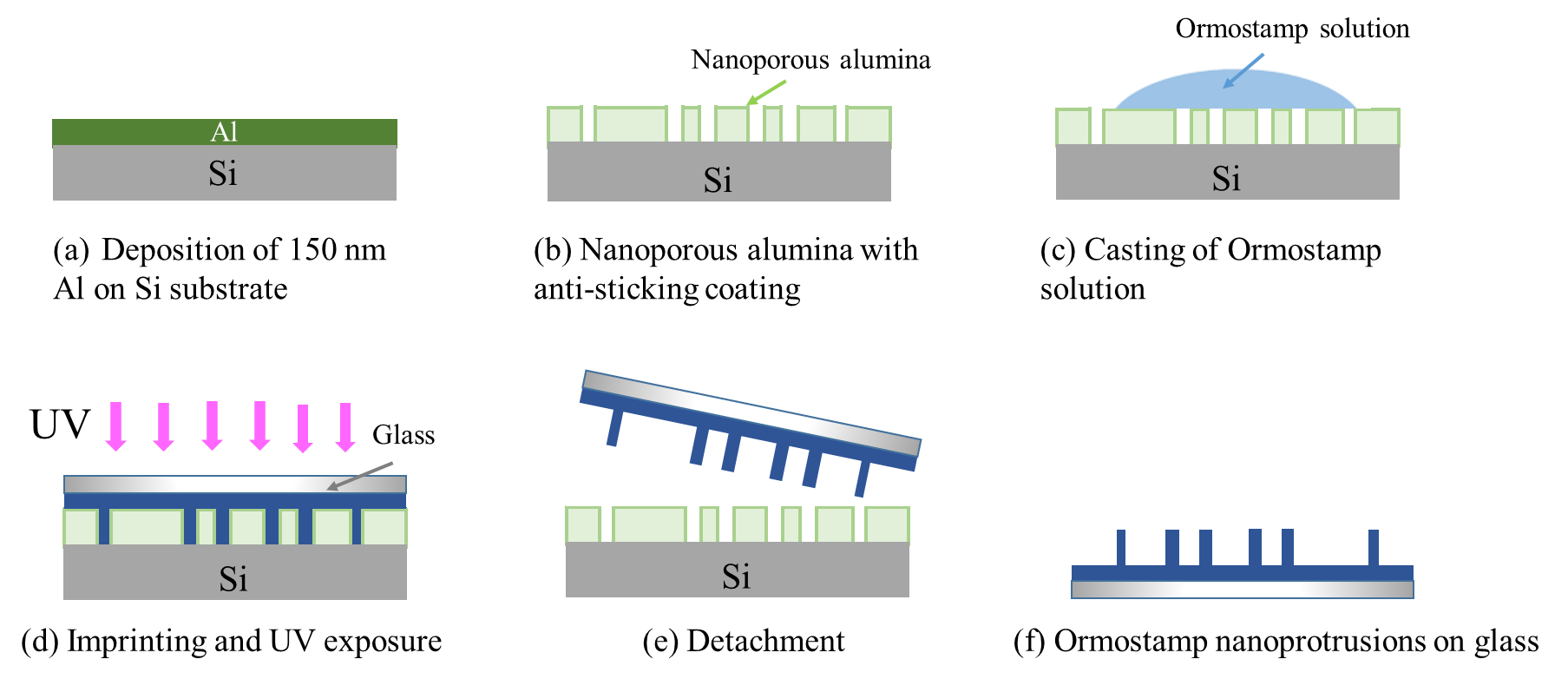
*

Figure S1a: Process flow of nanoimprint. (a) Preparation of nanopore template with anti-adhesive coating; (b) Casting of Ormostamp solution; (c) Imprinting with a glass cover slide, followed by UV curing; (d) Detachment from the nanopore template; (e) Ormostamp nanoprotrusion surfaces are obtained.


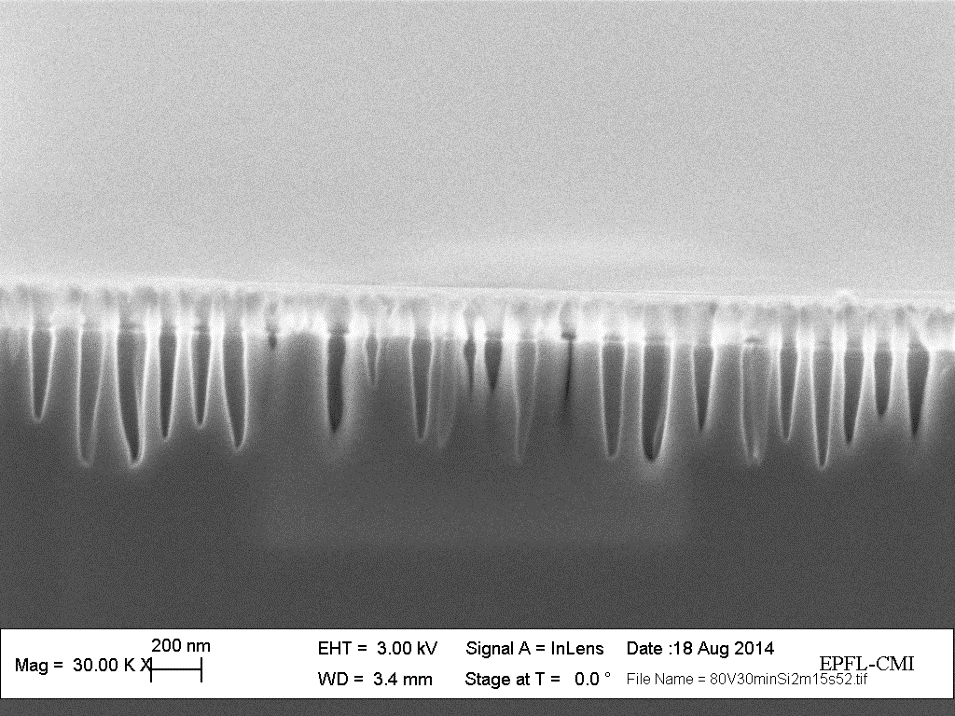


**Si wafer**

Alumina nanopore layer

Deep pores

Shallow pores

No obvious pore In Si layer

Figure S1b: Cross section image of a nanopore template after 3 min plasma etching of Si. This figure shows that more than half of the nanopore structures were etched into bottom Si wafer after 3 min deep reactive etching of Si through top alumina nanoporous layer. Deep and shallow trenches were observed in the Si layer. There are also some pore structures in the top layer which were not etched into the Si substrate, leading to reduced overall pore density in the Si layer. From this template, the nanostructured surface shown in **Fig. 1c** was obtained. After selectively dissolving the top alumina nanoporous layer (the layer above the dashed line), the nanostructure shown in **Fig. 1d** was obtained. The templates for fabricating Ormostamp nanostructured surfaces shown in **Fig. 1a-f** are schematically presented in following Fig. S1c.

Large connected cavities → Formation of Au nanonugget

Figure S1c: Schematics of various nanoporous templates. (a) With 1 min deep-reactive ion etching of Si through top nanoporous alumina layer; (b) nanoporous alumina; (c) With 3 min deep-reactive ion etching of Si through top nanoporous alumina layer; (d) Nanoporous Si template after selective dissolution of alumina layer shown in structure (c); (e) With 2 min deep-reactive ion etching of Si through top nanoporous alumina layer; (f) Nanoporous Si template after selective dissolving of alumina layer shown in structure (e). By using these templates, Ormostamp surfaces with nanopillars of various densities and heights are fabricated. The corresponding nanostructured Ormostamp surfaces are shown in **Fig. 1 a-f**.

2. Fluorescence image of *S. aureus* cells on smooth control surface.

Figure S2: Viability of *S. aureus* cells on smooth control Ormostamp surfaces analyzed by fluorescence microscopy after SYTO9/PI staining. Scale bar 20 μm.

3. Quantification of bactericidal efficiency by proliferation measurement

As described previously, the antibacterial efficiency of the reference and nanostructured surfaces with area of 1×1 cm^2^ was quantitatively assessed and compared using a proliferation assay [[1](#_ENREF_1)]. After cell attachment, the material samples were rinsed twice and incubated in fresh medium. After 2 h incubation, the supernatant was taken for turbidity measurements to monitor the proliferation of the attached cells. It is an indirect method to quantify the bactericidal ability of the surface nanostructures. The less viable cells adhered on sample surfaces, the longer time is needed to reach a specific OD value. Regardless of large error bars, it can be seen clearly 3 pairs of curves, corresponding to 3 typical pillar densities. S(c) and S(d) with pillar density at ~ 40 pillars μm^-2^ demonstrate longest time needed to reach a defined OD and therefor most efficient antibacterial property.


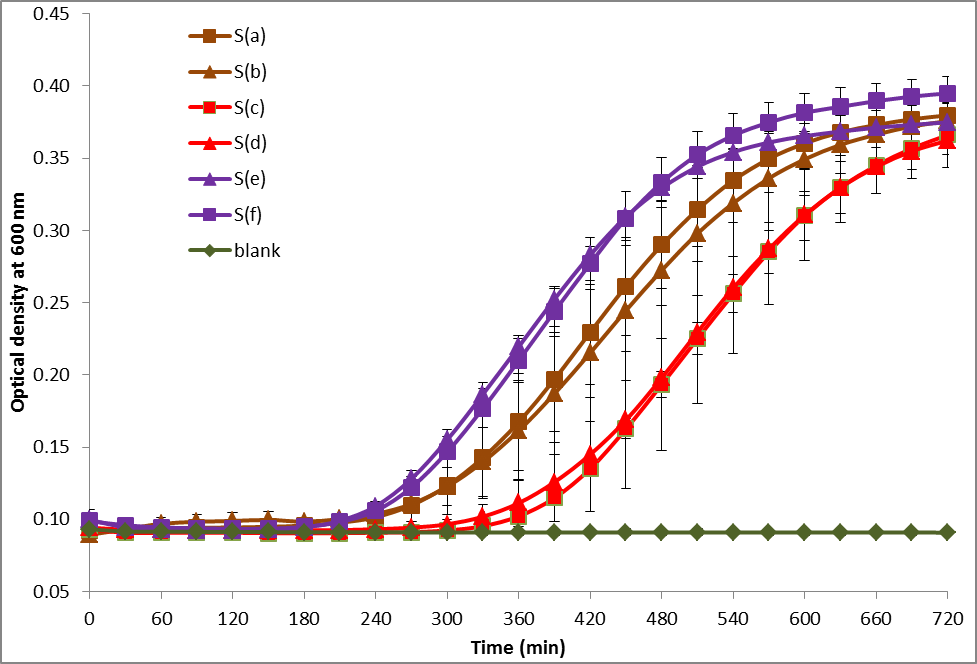


Figure S3: Proliferation curves of bacteria adhered onto nanostructured samples (S(a) – S(f)) with 1×1 cm^2^ surface area. Two independent experiments with three repeats per sample per experiment were performed. Error bars are shown as standard deviations for at least three measurements.

The obtained large error bars can be explained as follows: This assay is based on the time needed for the re-growth of the adhered cells in the presence of fresh medium. The time needed to start re-growth depends on the number of viable cells and their physiological condition (completely killed and dead, partially dead and growth can be recovered through cell repair systems, and fully viable). It can be expected that the physiological stage of the same bacterial preparation can vary slightly between the technical repeats of the same sample, caused by intrinsic heterogeneity of bacterial cultures. The variations in bacterial culture, along with the statistical error of the material surfaces can propagate to large variation of the measured time needed for re-growth of bacteria on the same samples. Indeed, high standard deviations for values generated from biological samples are often observed, compared to samples prepared by e.g. chemical or physical approach.

4. SEM images of *S. aureus* cells on nanostructured Ormostamp surfaces S(a)-S(f)

Typical SEM images of *S. aureus* cells on all the samples shown in Figure S4. Although the interaction of the cells with nanopillars can be noticed on all the nanostructures, obvious cell deformation can only be observed on structure S(c) and S(d) with effective pillar density of ~40 pillars μm^-2^. The bacterium is initially suspended on the nanopillar arrays so the deformation of the cell membrane is expected. Further deformation and retraction of the cell membrane may occur during the dehydration process when the sample is taken out of aqueous solution and placed under vacuum condition of SEM. As the bacterial cell is not an ideal sphere, the difference between the top and tilted views is sometimes limited.


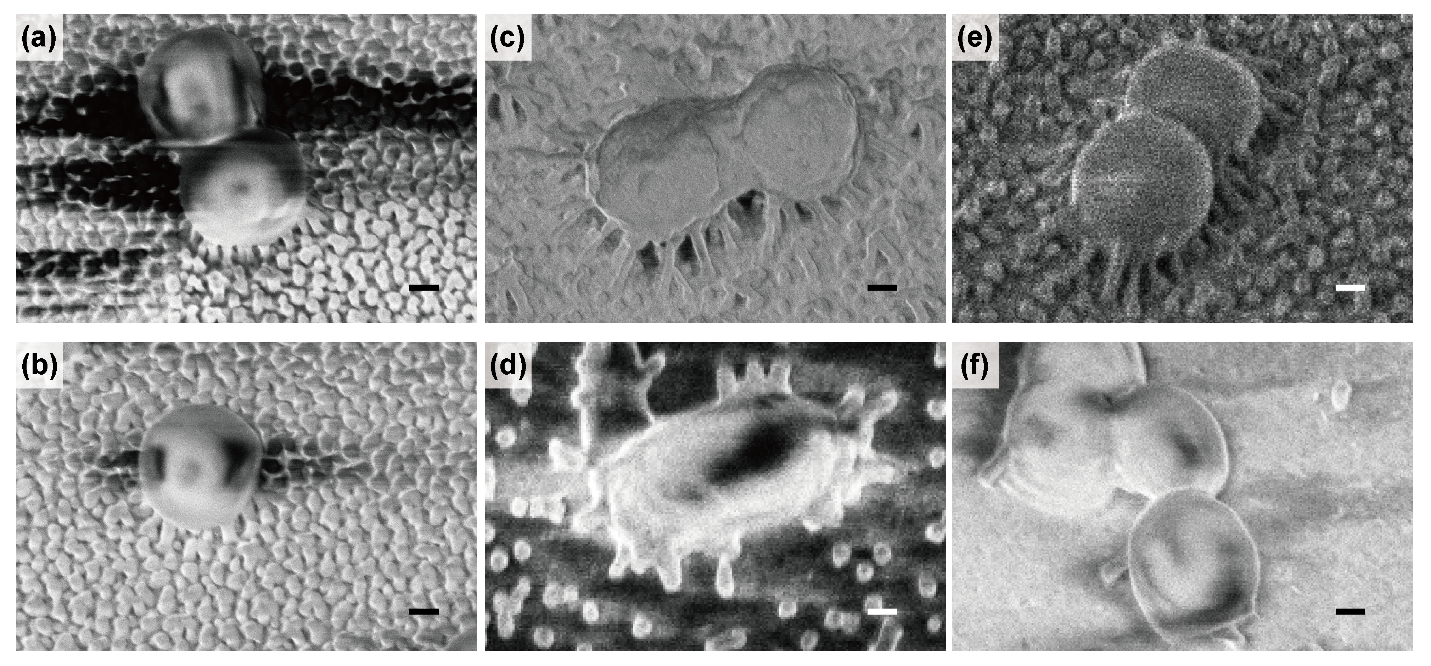


Figure S4. SEM images of *S. aureus* cells on nanostructured Ormostamp surfaces S(a)-S(f). Scale bar: 200 nm; Tilted angle: 30°.

5. Biophysical model

Figure S5. Biophysical model of bacterial cells adhered on nanostructured surfaces.

In the biophysical model described by S. Pogodin *et al*. [[2](#_ENREF_2)], the bacterial cell was considered as an elastic membrane adsorbed on nanopillar arrays with the stretching degree α_A_ on the pillars and α_B_ between them. Each site that is adsorbed on the nanopillar surface contributes the energy gain, which is balanced by the free-energy loss associated with deformation of the membrane. The total free energy F is


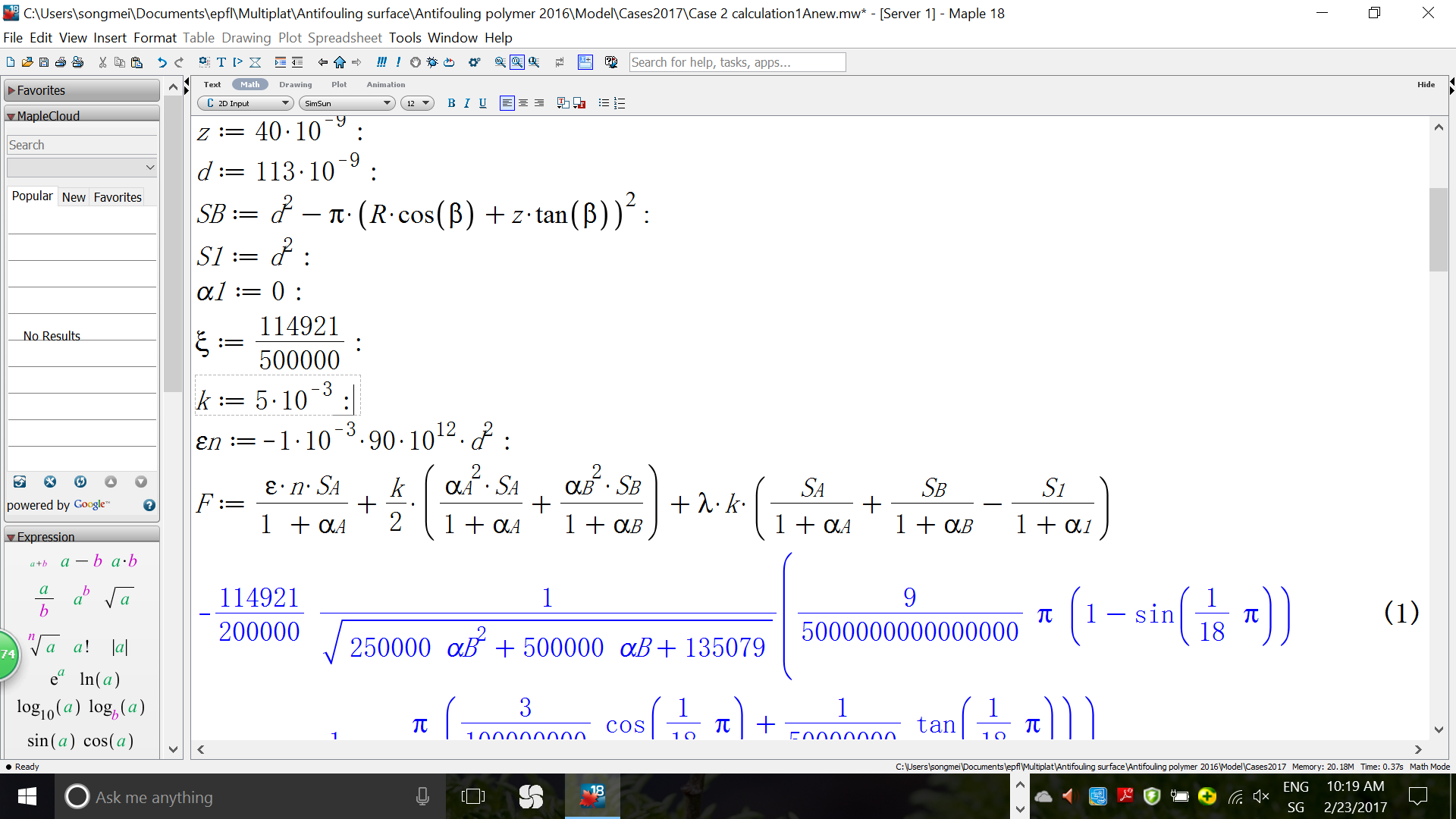


where Stretching modulus: k = 5×10^-3^ N m^-2^, Energy gain of adhesion: ε = −1×10^-3^ J m^-2^, Pillar density n: number m^-2^, Energy gain adhesion per site (take half sphere R = 30 nm): = −1×10^-3^ J m^-2^•pillar density n•d^2^, S_A_ is membrane area in contact with nanopillars and S_B_ is that suspending between nanopillars, S_1_ is the total initial area of unperturbed membrane = d^2^. Lagrange multiplier λ= ½ [(1+α_B_)^2^ − 1], with ξ = − ε n k^-1^.

S_A_ and S_B_ are calculated based on the geometry in Case I and Case II. The equilibrium stretching α_A_ and α_B_ of the membrane were obtained by numerical minimization of the total free energy F.

References:

[1] S. Wu, F. Zuber, J. Brugger, K. Maniura-Weber, Q. Ren, Antibacterial Au nanostructured surfaces, Nanoscale 8 (2016) 2026-2025.

[2] S. Pogodin, J. Hasan, V.A. Baulin, H.K. Webb, V.K. Truong, T.H.P. Nguyen, V. Boshkovikj, C.J. Fluke, G.S. Watson, J.A. Watson, R.J. Crawford, E.P. Ivanova, Biophysical model of bacterial cell interactions with nanopatterned cicada wing surfaces, Biophys. J. 104 (2013) 835-840.
